# New Evidence on Causal Relationship between Approximate Number System (ANS) Acuity and Arithmetic Ability in Elementary-School Students: A Longitudinal Cross-Lagged Analysis

**DOI:** 10.3389/fpsyg.2016.01052

**Published:** 2016-07-12

**Authors:** Yunfeng He, Xinlin Zhou, Dexin Shi, Hairong Song, Hui Zhang, Jiannong Shi

**Affiliations:** ^1^Key Laboratory of Behavioral Science, Institute of Psychology, Chinese Academy of SciencesBeijing, China; ^2^National Key Laboratory of Cognitive Neuroscience and Learning, Beijing Normal UniversityBeijing, China; ^3^Department of Psychology, University of OklahomaNorman, OK, USA

**Keywords:** approximate number system, math performance, arithmetic ability, elementary-school students, cross-lagged analysis, longitudinal study

## Abstract

Approximate number system (ANS) acuity and mathematical ability have been found to be closely associated in recent studies. However, whether and how these two measures are causally related still remain less addressed. There are two hypotheses about the possible causal relationship: ANS acuity influences mathematical performances, or access to math education sharpens ANS acuity. Evidences in support of both hypotheses have been reported, but these two hypotheses have never been tested simultaneously. Therefore, questions still remain whether only one-direction or reciprocal causal relationships existed in the association. In this work, we provided a new evidence on the causal relationship between ANS acuity and arithmetic ability. ANS acuity and mathematical ability of elementary-school students were measured sequentially at three time points within one year, and all possible causal directions were evaluated simultaneously using cross-lagged regression analysis. The results show that ANS acuity influences later arithmetic ability while the reverse causal direction was not supported. Our finding adds a strong evidence to the causal association between ANS acuity and mathematical ability, and also has important implications for educational intervention designed to train ANS acuity and thereby promote mathematical ability.

## Introduction

### Theoretical Background

Approximate Number System (ANS) underlies the ability to approximately represent numbers without verbal counting or the involvement of numerical symbols. ANS is shared across species, not only in humans including preverbal infants (McCrink and Wynn, 2004; Xu et al., 2005), but also in non-human animals, such as monkeys (Flombaum et al., 2005; Cantlon and Brannon, 2006). It allows preverbal human infants to perceive and discriminate numerosities, and persists over the lifespan of humans (Halberda and Feigenson, 2008; Halberda et al., 2012). Beyond the ANS, humans have also acquired the unique precise symbolic representation system and mathematical abilities through explicit instructions (e.g., Butterworth, 1999; Nieder and Dehaene, 2009).

Recently, these two number representation systems and the corresponding abilities, i.e., ANS acuity and mathematical ability, have been found to be closely related (see a review by Chen and Li, 2014). Halberda et al. (2008) reported that individual differences of ANS acuity of 14-year-old children were retrospectively related to their standardized math scores from kindergarten to sixth grade. Following this work, various works have revealed that individual differences of pre-schooled children in ANS acuity are associated with their current (Inglis et al., 2011) and future mathematic achievements (Gilmore et al., 2010; Piazza et al., 2010; Libertus et al., 2011, 2012; Mazzocco et al., 2011; Starr et al., 2013). As an example, Libertus et al. (2012) showed that the ANS acuity of college students correlates with their scores on Scholastic Aptitude Test (SAT) even after controlling for verbal abilities. However, inconsistent findings were also reported. Sasanguie et al. (2014) found that ANS acuity of kindergarteners does not predict their performances on symbolic comparison tasks six months later. Moreover, several researches showed that the relationship between ANS acuity and math performance is mediated by inhibitory control (Fuhs and McNeil, 2013) or visual perception (Zhou and Cheng, 2014; Zhou et al., 2015).

Although the correlations between ANS acuity and mathematical achievement have been repeatedly reported in many studies, the causal direction of this association, if exists, still remains inconclusive. Two contrary hypotheses about the causal direction of this association were reported in the literature. One is that ANS acuity causally influences later mathematical achievements, i.e., children with higher ANS acuity tend to perform better in arithmetic calculations and high-level mathematics. In support of this hypothesis, several recent studies found that individual differences in ANS acuity before introduction of formal education predict later mathematic achievements (Libertus and Brannon, 2010; Piazza et al., 2010; Desoete et al., 2012; Starr et al., 2013). Moreover, there are studies showing that simple practice or training on ANS tasks improves later arithmetic performance both in adults and children (Park and Brannon, 2013; Hyde et al., 2014). Another hypothesis suggests that access to math education sharpens the ANS acuity. Main evidence supporting the second hypothesis comes from the comparison between ANS acuity of individuals with and without access to math education (Nys et al., 2013; Piazza et al., 2013; Lindskog et al., 2014). For instance, by comparing the ANS acuity of the members of an indigene Mundurucu group with highly variable access to mathematics education, Piazza et al. (2013) showed that individuals taking math education have significantly higher ANS acuity compared to their unschooled peers.

### Research Question

Evidence seems to arise in support of both hypotheses. However, we notice that due to experimental constraints, in every aforementioned individual study only one causal direction of the association between ANS and mathematic ability was tested, while leaving the reverse one or reciprocal effect untested. Therefore, questions still remain whether only one causal direction or reciprocal causal effects existed in the association of ANS acuity and mathematical ability. An simultaneous assessment of all potential causal directions of the association between ANS acuity and mathematical ability was therefore required.

## Materials and Methods

To further probe the possible causal relationships existed between ANS acuity and mathematical ability, the present study longitudinally collected data on ANS acuity and arithmetic ability of elementary-school students at three time points. These data were analyzed using a cross-lagged regression analysis, which allows all potential causal directions to be evaluated simultaneously. Four competing cross-lagged models correspond to four patterns of causal effects, i.e., no causal effect, causal effect from ANS acuity to arithmetic ability, causal effect from arithmetic ability to ANS acuity, and reciprocal causal effects (Burkholder and Harlow, 2003; Creed et al., 2006). By this longitudinal cross-lagged method, the assessment of all possible causal directions of the association between ANS acuity and arithmetic ability was therefore possible.

### Participants

Participants were Grade 3, Grade 4, and Grade 5 students from a primary school in Beijing, about 50 students for each grade. Their demographic information is summarized in **Table 1** These participants were first tested when they were at the end of their grades and then retested every 6 month in the following year. As shown in **Table 1**, there were overall 162, 144, and 136 students participating in the tests conducted at Time 1 (T1), Time 2 (T2), and Time 3 (T3), respectively. Compared to T1, there were 18 and 26 students dropped out of the ANS and arithmetic tasks at T2 and T3, respectively. The drop out of participants at T2 and T3 were mainly due to sick leave or school transfer. The 6-month interval between data collection points were considered to be long enough for possible changes in ANS acuity and arithmetic ability and not too long in order to avoid losing a large number of participants at T2 and T3. The chi-square analysis shows that there were no significant differences in terms of the gender of participants at T1, T2, and T3 (*χ*^2^ (4) = 6.000, *p* = 0.199).

**Table 1:** Demographic information of participants.

Characteristics	T1	T2	T3
	*M* (*SD*) or *n* (%)	*M* (*SD*) or *n* (%)	*M* (*SD*) or *n* (%)
Total number	162	144	136
Gender
Female	71 (43.80)	61 (42.40)	56 (41.20)
Male	91 (56.20)	83 (57.60)	80 (58.80)
Age	10.33 (0.99)	10.80 (0.91)	11.32 (0.91)

Before participation, all children and their parents signed informed consent forms. There were no vulnerable populations involved in the current study. The study was approved by the Ethics Committee of the Institute of Psychology, Chinese Academy of Sciences.

### Experimental Tasks

#### ANS Acuity Task

**FIGURE 1:**
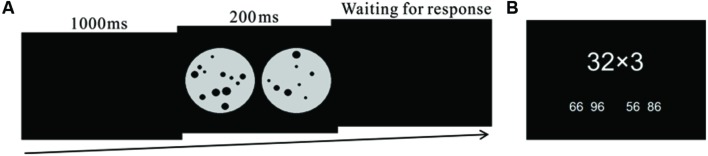
**(A)** Schematic of the dot-array comparison task used to measure Approximate Number System (ANS) acuity of participants. **(B)** An example of multiplication task used to measure arithmetic ability of participants.

The ANS acuity of the participants was assessed by a non-symbolic dot comparison task, adapted from the second edition of the Test of Early Mathematics Ability (Ginsburg and Baroody, 1983). The task has been used in recent studies (Zhou and Cheng, 2014; Zhou et al., 2015). As shown in **Figure 1A**, three screens were presented sequentially in this task. Screen 1 and Screen 3 were black screens containing no information; on Screen 2, two sets of dot-arrays with different number of dots were presented. The participants were asked to choose the more numerous dot-array by pressing the corresponding keys on the computer keyboard. The dot-arrays were created following a common procedure to control for the non-numerical, continuous variables in the ANS task (e.g., Halberda et al., 2008; Agrillo et al., 2013). For half of the ANS tasks, the total combined area of all dots in each dot-array was the same; for the other half of the ANS trials, the average area of dots in each set was the same. The dots in each dot-array were randomly distributed within a circle and the sizes of the dots varied randomly. The number of dots in each dot-array varied from 5 to 32, and the ratio between the number of dots in the two dot-arrays was above 1.2 but below 2.0. The second screen with dot-arrays was only presented for 200 ms, which was too short for participants to count the dots individually. There was no time limit for an answer. The test consisted of 120 trials, with 40 trials for each session. The ANS acuity of the participants was measured using the accuracy, rather than other indices, as suggested by Inglis and Gilmore (2014). The Cronbach’s alpha of the ANS acuity task was 0.80 in the current study.

#### Arithmetic Ability Task

The arithmetic ability of participants was assessed using multiplication tasks with the form of one single-digit number multiplied by one two-digit number such as “6 × 15”, as did in previous study (Wei et al., 2012). Accompanying each multiplication question, four candidate answers were presented, and the participants were asked to choose which one is the correct by pressing corresponding keys on a computer keyboard. The three incorrect candidate answers were constrained to be the correct answer ±1–100. For example, for the question of 3 × 32, the four optional answers were 66, 96, 56, and 86 (**Figure 1B**). There were a total number of 76 multiplication questions administered within two minutes in this task and there was 1 s between each trial. On average, the participants completed 34.8, 38.0, and 41.1 multiplication tasks at T1, T2, and T3, respectively. Accordingly, the average time spent on each multiplication task by the participants was about 2.4, 2.2, and 1.9 s at T1, T2, and T3, respectively.

The performances of the participants were measured using the scores calculated by Guilford correction formula *S* = *R* -*W*/(*N* - 1), where *S* is the adjusted number of items that the participants can actually perform without the aid of guessing, *R* is the number of correct responses, *W* is the number of incorrect responses, and *N* is the number of alternative responses to each item (Guiford, 1936; Cirino, 2011). The participants were told to calculate fast and accurately. They were not informed about whether they can use guessing or not. It should be noted that the arithmetic ability task used in the current study is time limited (2 min), and the number of questions far exceeded what can be answered by participants. In consequence, participants may use guessing to answer the calculation questions. Indeed, the participants were observed to guess, especially at the end of the task. Therefore, in order to control for the effect of guessing, the Guilford correction formula was used, as did in many previous studies (Salthouse, 1994; Putz et al., 2004; Hedden and Yoon, 2006; Cirino, 2011; Wei et al., 2012). The split-half reliability of the arithmetic ability test was 0.90. No pencils or papers were allowed during the whole test. Both ANS acuity and arithmetic ability tasks were computerized using web-based applications in the “Online Psychological Experiment System (OPES)”.^1^

### Procedure

For each group of participants, the ANS acuity task and arithmetic ability task were conducted sequentially with 5 min break between. Completion of two tasks (including the 5 min break) usually takes less than 20 min. Every group consisted of 20–26 participants, and for every 4–6 participants, one experimenter was assigned to ensure that participants paid full attention to the tasks. Practice trials were conducted before the formal task to ensure that all participants understood the tasks. For ANS acuity and arithmetic ability tasks, four and six practice trials were conducted, respectively. The practice trials were similar but easier compared to those used in the formal test. The children could ask any questions during the practice session and was then instructed by the experimenter. After all the participants had finished the practice session and had no more questions, the formal tests were then conducted.

### Data Analysis

**FIGURE 2:**
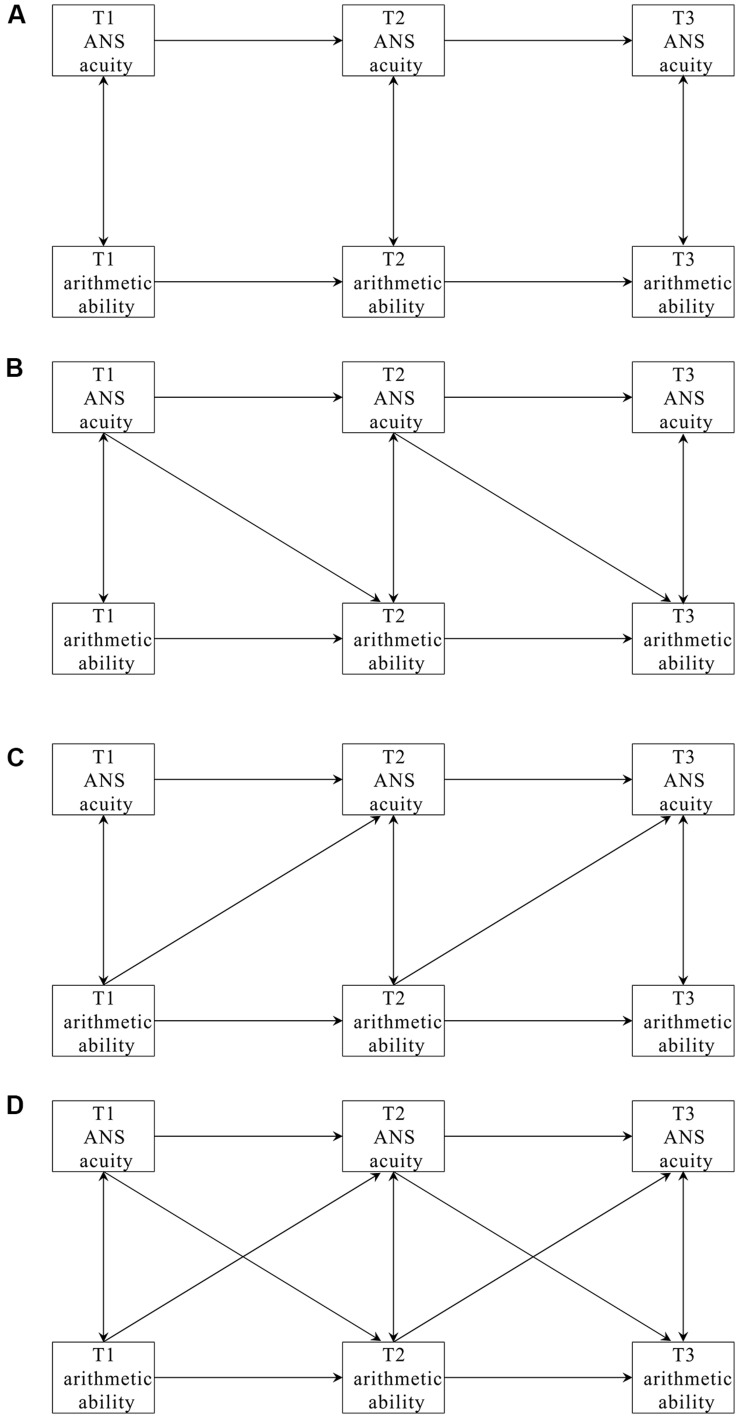
**Four-cross-lagged models used to fit the data. (A–D)** Refer to Model 1, Model 2, Model 3 and Model 4, respectively.

Three-wave longitudinal panel data were collected on ANS acuity and arithmetic ability of the participants at three sequential time points. Prior to cross-lagged regression analysis, the descriptive statistics and the correlations of these two variables were first computed by SPSS 20.0. Mplus 7.0 was then used to fit the four competing cross-lagged models to the collected data, in order to test the causal relationships between ANS acuity and arithmetic achievement (**Figure 2**). The first model (M1) was an autoregressive model, with no cross-lagged effects but only temporal stability and contemporary associations. The second model (M2) added cross-lagged pathways from ANS acuity at T1 (and T2) to arithmetic ability at T2 (and T3), testing the hypothesis that ANS acuity has a causal effect on arithmetic achievement. The third model (M3) added the cross-lagged pathways from arithmetic ability at T1 (and T2) to ANS acuity at T2 (and T3), testing the hypothesis that arithmetic education would enhance ANS acuity. The last model (M4) represented reciprocal effects and tested causal effects in both directions. In the current study, considering that there were only three waves of data, the time-specific effects of cross-lagged paths were not explored; instead, the two cross-lagged paths from T1 to T2 and T2 to T3 of M2 were constrained to be equal, which indicates the average effects of ANS acuity on arithmetic ability across one-year period. Similar equality constrains were used in M3 and M4 as well.

Model fit of four competing cross-lagged models to our data were mainly assessed by four indices, i.e., Chi-square statistic (*χ*^2^), Tucker–Lewis Index (TLI), Comparative fit index (CFI) and Root-mean-square error of approximation (RMSEA). Chi-square statistic was used as the primary criteria to evaluate model fit, with the smallest value meaning the best model fit. TLI and CFI range from 0 to 1, with values greater than 0.90 indicating adequate model fit (Hu and Bentler, 1999). When RMSEA is less than or equal to 0.05, it indicates close approximation; when RMSEA is between 0.05 and 0.08, it indicates a reasonable error of approximation; when RMSEA is between 0.08 and 0.10, it provides a mediocre fit; when RMSEA is greater than or equal to 0.10, it means poor fit (Browne et al., 1993).

## Results

### Attrition Analysis

There were 162 children participating at the ANS acuity and arithmetic ability tasks at T1, while only 144 and 136 completed the same tasks at T2 and T3. Compared to T1, 18 students dropped out of tests conducted at T2, and compared to T2, another 8 students dropped out of the tests conducted at T3. The missing data for each participant were imputed using FIML. The analysis shows that the 18 dropouts did not significantly influence the ANS acuity data at T1 (*t*(160) = -0.95, *p* = 0.343), or arithmetic ability data at T1 (*t*(160) = -1.55, *p* = 0.123). Neither did 8 dropouts significantly change the ANS acuity data at T2 (*t*(142) = -0.53, *p* = 0.597), or arithmetic ability data at T2 (*t* = -0.124, *p* = 0.901).

### Correlation between ANS Acuity and Arithmetic Ability

Before cross-lagged regression analysis, the means, standard deviations and correlations of ANS acuity and arithmetic ability at T1, T2, and T3 were first calculated (**Table 2**). It was shown that the ANS acuity of participants increases gradually with ages, and shows significant differences between any two different time points during the testing period (*F*(2) = 39.79, *p* < 0.001). The arithmetic ability of participants also increases with ages, but only shows significant difference between T1 and T2, T1 and T3 (*F*(2) = 3.84, *p* < 0.05). Moreover, the correlation matrix (**Table 2**) shows that there were strong correlations between ANS acuity and arithmetic ability during the test period. Notably, the strength of the contemporary correlations of ANS acuity with arithmetic ability remained almost unchanged (0.33∼0.34, *z* = 0∼0.15, *ps* > 0.05, **Table 2**) during the age period roughly around 10–12 years-old.

**Table 2:** Means (*M*), standard deviations (*SD*), and correlation matrix of Approximate Number System (ANS) acuity and arithmetic ability of participants at three time points (T1, T2, and T3).

Measures	*M*	*SD*	1	2	3	4	5
1. ANS acuity (T1)	56.66	2.23	–				
2. ANS acuity (T2)	66.70	2.01	0.62^∗∗∗^	–			
3. ANS acuity (T3)	72.48	1.44	0.48^∗∗∗^	0.63^∗∗∗^	–		
4. Arithmetic ability (T1)	26.00	0.66	**0.33**^∗∗∗^	0.26^∗∗^	0.25^∗∗^	–	
5. Arithmetic ability (T2)	27.38	0.55	0.17^∗^	**0.34**^∗∗∗^	0.17^∗^	0.47^∗∗∗^	–
6. Arithmetic ability (T3)	27.53	0.56	0.35^∗∗∗^	0.34^∗∗∗^	**0.33**^∗∗∗^	0.49^∗∗∗^	0.43^∗∗∗^

**^∗^p < 0.05. ^∗∗^p < 0.01. ^∗∗∗^p < 0.001.* The contemporary correlation coefficients between ANS acuity and arithmetic ability were shown in bold*.

### Test of Cross-Lagged Models

**FIGURE 3:**
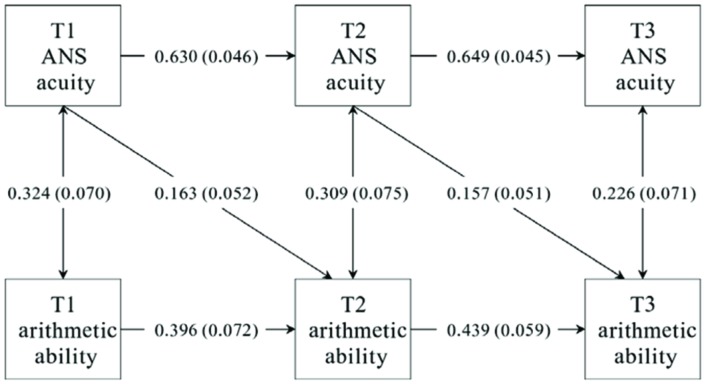
**The fitted optimal cross-lagged model (M2) with standardized path coefficients.** The numbers attached to the arrows were standardized path coefficients, with *sig* errors in the brackets.

**Table 3** summarizes the fit indices of the four competing cross-lagged models, i.e., M1 (with no cross-lagged effect), M2 (with cross-lagged effect from ANS acuity to arithmetic ability), M3 (with cross-lagged effect from arithmetic ability to ANS acuity), and M4 (with reciprocal cross-lagged effects). The four indices suggest that M1 demonstrates a good fit to the data (χ^2^ (8) = 23.456, TLI = 0.904, CFI = 0.945, RMSEA = 0.098). Furthermore, by adding the cross-lagged path from ANS acuity to arithmetic ability, M2 shows significant improvement of fit to the data compared to M1 (Δχ^2^ (1) = 9.624, *p* < 0.001); in contrast, M3 shows no significant improvement when adding the cross-lagged paths from arithmetic ability to ANS acuity (Δχ^2^ (1) = 0.766, *p* = 0.381). Furthermore, a close comparison between M4 and M2 shows that adding the cross-lagged paths from arithmetic ability to ANS acuity does not improve the fit of M2 to the data (Δχ^2^ (1) = 0.324, *p* = 0.569). Therefore, the cross-lagged effect from arithmetic ability to ANS acuity was not supported. M2 was selected as the best-fitted model, and the standardized regression coefficients of M2 are shown in **Figure 3**

**Table 3:** Summary fit statistics for the four cross-lagged models.

Model	*χ*^2^ (*df*)	TLI	CFI	RMSEA
Non cross-lagged (M1)	23.456 (8)	0.904	0.945	0.098
Cross ANS→Arithmetic (M2)	13.832 (7)	0.951	0.976	0.070
Cross Arithmetic→ANS (M3)	22.690 (7)	0.888	0.944	0.105
Both Cross (M4)	13.508 (6)	0.938	0.973	0.079

**Notes*: χ^*2*^= chi-square; TLI = Tucker-Lewis Index; CFI = Comparative Fit Index;RMSEA = Root Mean Square Error ofApproximation; M1 = Model 1; M2 = Model 2; M3 = Model 3; M4 = Model 4. M1 represents temporal stability and contemporary associations and has no cross-lagged effects; M2 and M3 represent a causal direction from ANS acuity to arithmetic ability and the reverse direction, respectively; M4 represents mutual causal directions between ANS acuity and arithmeticability*.

## Discussion

In the present study, we tracked the development of ANS acuity and arithmetic ability of elementary-school students during one-year period. Strong correlations were found between ANS acuity and arithmetic ability of the students, which is consistent with previous results obtained in other age periods (Halberda et al., 2008; Gilmore et al., 2010; Piazza et al., 2010; Inglis et al., 2011; Libertus et al., 2011, 2012; Mazzocco et al., 2011; Starr et al., 2013; Chen and Li, 2014). More importantly, by estimating the fit of four competing cross-lagged models to the collected data, we tested four hypotheses about the causal relationships between ANS acuity and arithmetic ability simultaneously. The results show that M2 with the cross-lagged pathway from ANS acuity to arithmetic ability fits the best, while other cross-lagged models with reverse or reciprocal pathways fail to fit the data. That means, ANS acuity of the participants at T1 predetermines their arithmetic ability at T2, and the ANS acuity of the participants at T2 further determines their arithmetic performance at T3.

The results presented here were consistent with previous studies suggesting that ANS acuity causally influences arithmetic ability (Libertus and Brannon, 2010; Piazza et al., 2010; Desoete et al., 2012; Park and Brannon, 2013; Starr et al., 2013; Hyde et al., 2014). All these previous studies point to the same suggestion that ANS acuity has a causal influence on arithmetic ability, although the reverse causal effect was left untested. Therefore, the present study provides a more systematic estimation of the causal relationships between ANS acuity and arithmetic ability, and adds a strong piece of evidence that ANS acuity has a causal effect on the future arithmetic achievements. Moreover, the established causal direction between ANS acuity and arithmetic ability also suggests important implications for educational intervention, which could be designed to sharpen the ANS acuity of children and thereby promote their future mathematical abilities. Besides, the lack of causal influence of math education on ANS acuity as presented in this work was consistent with some previous studies (Castronovo and Göbel, 2012; Zebian and Ansari, 2012).

However, here it must be emphasized that the present study only investigated the causal relationship between ANS acuity and arithmetic ability in elementary-school students, aged around 10–12 years-old. In other age periods or population groups, the causal direction between ANS acuity and arithmetic ability may be different, and reversed causal direction or even reciprocal casual effect might exist. Indeed, several recent studies found that access to math education can improve ANS acuity (Nys et al., 2013; Piazza et al., 2013; Lindskog et al., 2014). Actually, the inconsistent results not only exist in the causal direction of the association between ANS acuity and arithmetic ability, but also in whether this association exists (De Smedt et al., 2013; Chen and Li, 2014). At this moment, it is still very hard, even impossible, to conclude what leads to the inconsistent findings. As far as we know, three factors may partially account for the inconsistent results. The first factor is the demographic features of population, such as age. The causal direction of the association between ANS acuity and arithmetic ability may change under different periods of human development (Inglis et al., 2011). While our study was based on elementary-school students, aged around 10–12 years-old, several studies reporting a causal direction from arithmetic ability to ANS acuity drew their conclusion from the study of adults (Nys et al., 2013; Lindskog et al., 2014). The second factor is the methodology used to measure ANS acuity and arithmetic ability. The tasks used to measure ANS acuity in the literature are highly different (Price et al., 2012), and as pointed by Dietrich et al. (2015), the difference of reliability in ANS tasks leads to inconsistent results on the association between ANS acuity and arithmetic ability. The third one is potential moderators (De Smedt et al., 2013). For example, inhibitory control (Gilmore et al., 2013) and visual perception (Zhou et al., 2015) may account for the relationship between ANS acuity and arithmetic ability. A systematic study of the influences of these factors on the causal relationship between ANS acuity and arithmetic ability is needed to clarify what leads to the inconsistent findings in future studies.

Although the longitudinal cross-lagged analysis adds a strong evidence to the debate on the causal relationship between ANS acuity and arithmetic ability, it does not mean the causal direction between this association was guaranteed by this methodology. In this pilot study, only the data of ANS acuity and arithmetic ability of participants were measured while other covariates, such as intelligence, speed of processing and working memory, were not controlled. These covariates may have possible influences on the causal relationship between ANS acuity and arithmetic ability. Therefore, future research will need to measure and consider these potential variables when investigating the causal direction of the association between ANS acuity and arithmetic ability.

The mechanisms underlying the causal relationship between ANS acuity and arithmetic ability still remain unclear. Several explanations have been proposed to explain why ANS acuity influences later mathematical performances. One possibility is that children with more accurate ANS acuity might have more confidence when dealing with exact symbolic numbers at the very beginning, and the early-gained advantage may in turn lead to more engagement and investment into symbolic number-related issues and eventually promote their arithmetic abilities (Libertus et al., 2011; Halberda et al., 2012). A second possibility is that more accurate ANS might enable children to online check the possible errors occurred in arithmetic calculations (Lourenco et al., 2012) and therefore serves as an accessory system for symbolic functioning. A third possibility is that more accurate number sense might help children understand the concept of symbolic numbers, computing rules and the meanings of numerical words (Dehaene, 1997; Feigenson et al., 2013). This predicts that training on non-symbolic numerical addition and subtraction would help improve the performance on arithmetic calculations, as already evidenced by recent studies (Park and Brannon, 2013; Hyde et al., 2014). Besides, insights about the causal relationship between ANS acuity and arithmetic ability might be gained from neuro imaging methods (Nieder and Dehaene, 2009; De Smedt et al., 2013).

## Future Proposal

In conclusion, the longitudinal cross-lagged analysis could simultaneously assess all potential causal directions of the association between ANS acuity and arithmetic ability. It would be meaningful in future research to apply this methodology to other age periods of human development, and therefore identify how the causal relationship evolves with age. This might help decide the best time to train ANS acuity of students and thereby promote their math performance more efficiently. Another aspect that needs to be addressed in future study is to explore the mechanism underlying the causal direction from ANS acuity to math performance.

## Author Contributions

YH, XZ, and JS initiated the project and conceived the experiments. YH and XZ designed the ANS acuity and arithmetic ability tasks. YH and HZ performed the tests and collected the data. DS and HS used the cross-lagged model to fit the collected data and did the analysis. YH and JS wrote the manuscript. All authors contributed to the analysis of the results, revised the manuscript, approved the final version and agreed to be accountable for all aspects of the work.

## Conflict of Interest Statement

The authors declare that the research was conducted in the absence of any commercial or financial relationships that could be construed as a potential conflict of interest.

## Footnotes

1 http://www.dweipsy.com/lattice
